# Development of an unannounced standardized patient protocol to evaluate opioid use disorder treatment in pregnancy for American Indian and rural communities

**DOI:** 10.1186/s13722-021-00246-6

**Published:** 2021-06-25

**Authors:** A. Taylor Kelley, Marcela C. Smid, Jacob D. Baylis, Elizabeth Charron, Amy E. Binns-Calvey, Shayla Archer, Saul J. Weiner, Lori Jo Begaye, Gerald Cochran

**Affiliations:** 1 Informatics, Decision-Enhancement and Analytic Sciences (IDEAS) Center, VA Salt Lake City Health Care System, 500 Foothill Drive, Building 2, Salt Lake City, UT 84148 USA; 2 Program of Addiction Research, Clinical Care, Knowledge, and Advocacy (PARCKA), Division of Epidemiology, Department of Internal Medicine, University of Utah School of Medicine, 295 Chipeta Way, Salt Lake City, UT 84132 USA; 3 Division of General Internal Medicine, Department of Internal Medicine, University of Utah School of Medicine, 30 N 1900 E 5R341, Salt Lake City, UT 84132 USA; 4 Department of Obstetrics and Gynecology, University of Utah School of Medicine, 30 N 1900 E 2B300, Salt Lake City, UT 84132 USA; 5 Jesse Brown VA Medical Center, Medical Services, 820 S Damen Ave, Chicago, IL 60612 USA; 6 Division of Academic Internal Medicine and Geriatrics, Department of Medicine, University of Illinois At Chicago, 840 South Wood Street, CSN 440, Chicago, IL 60612 USA; 7 Edward Hines VA Hospital, Center of Innovation for Complex Chronic Healthcare, 5000 5th Avenue, Hines, IL USA

**Keywords:** Opioid use disorder, Pregnancy, Unannounced standardized patients, Rural health care, Access to care, Quality of care

## Abstract

**Background:**

Opioid use disorder (OUD) disproportionately impacts rural and American Indian communities and has quadrupled among pregnant individuals nationwide in the past two decades. Yet, limited data are available about access and quality of care available to pregnant individuals in rural areas, particularly among American Indians (AIs). Unannounced standardized patients (USPs), or “secret shoppers” with standardized characteristics, have been used to assess healthcare access and quality when outcomes cannot be measured by conventional methods or when differences may exist between actual versus reported care. While the USP approach has shown benefit in evaluating primary care and select specialties, its use to date for OUD and pregnancy is very limited.

**Methods:**

We used literature review, current practice guidelines for perinatal OUD management, and stakeholder engagement to design a novel USP protocol to assess healthcare access and quality for OUD in pregnancy. We developed two USP profiles—one white and one AI—to reflect our target study area consisting of three rural, predominantly white and AI US counties. We partnered with a local community health center network providing care to a large AI population to define six priority outcomes for evaluation: (1) OUD treatment knowledge among clinical staff answering telephones; (2) primary care clinic facilitation and provision of prenatal care and buprenorphine treatment; (3) appropriate completion of evidence-based screening, symptom assessment, and initial steps in management; (4) appropriate completion of risk factor screening/probing about individual circumstances that may affect care; (5) patient-directed tone, stigma, and professionalism by clinic staff; and (6) disparities in care between whites and American Indians.

**Discussion:**

The development of this USP protocol tailored to a specific environment and high-risk patient population establishes an innovative approach to evaluate healthcare access and quality for pregnant individuals with OUD. It is intended to serve as a roadmap for our own study and for future related work within the context of substance use disorders and pregnancy.

**Supplementary Information:**

The online version contains supplementary material available at 10.1186/s13722-021-00246-6.

## Background

Opioid use disorder (OUD) in pregnancy is a life-threatening condition and major public health concern in the US. From 1999–2014, the national prevalence of OUD among women hospitalized for childbirth quadrupled, from 1.5 to 6.5 per 1000 [1], and neonatal abstinence syndrome increased nearly fivefold over a similar period [2]. Drug-induced death has emerged as a leading cause of pregnancy-associated mortality, with a majority attributed to opioids [3, 4].

Of particular concern is that American Indian (AI) and rural-dwelling populations, who are disproportionately impacted by OUD, are less likely to have access to treatment [5–13]. Additionally, with few specialists and opioid treatment programs available in rural communities [14, 15], ensuring quality among frontline primary care-based providers treating OUD in pregnancy is critical. This is particularly true for office-based buprenorphine treatment, which allows for sustainable delivery of OUD treatment within existing rural healthcare infrastructure [16, 17].

Little is known about how pregnant individuals with OUD access care within rural healthcare settings or the quality of care they receive. One primary reason for this knowledge gap is the difficulty of defining and measuring access and quality with administrative data, chart review, or qualitative interviewing. For example, whether and how healthcare services are facilitated for pregnant individuals with OUD is generally considered an important indicator of access but cannot be readily measured through these approaches [18]. Reports in medical records or administrative data may also vary from actual delivery of care.

Unannounced standardized patients (USPs)—a type of “secret shoppers” assigned standardized characteristics to compare observed care to expected care and test interventions—have been used increasingly to address gaps in the evaluation of access and quality for primary and specialty care [19–33]. The USP approach allows for *intrinsic risk adjustment*—an ability to control for potential confounding patient characteristics by standardizing those characteristics in actors portraying real patients—in order to test hypotheses for specific outcomes [18, 29, 34]. A single outcome may be observed across sampled entities (e.g., appointment wait times) as a pre-experimental design, or an intervention may be compared to a control (e.g., appointment wait times for AI compared to white patients) as an experimental design. Direct observation by USPs both enables capture of data not recorded in written records and avoids confounders, such as recall bias in qualitative interviewing and reporting bias in administrative data/chart review [35]. Because USP studies are blinded to the organizations or subjects being studied, they are not subject to the Hawthorne effect [30].

One recent telephone-based USP study among pregnant women with OUD demonstrated that significant barriers and high out-of-pocket costs are often present when seeking care from an OUD treatment provider [36]. However, to our knowledge, high-risk patient populations, such as AI and rural communities, have not been targeted in any previous such analyses. We therefore sought to address this gap by developing a hypothesis-testing USP protocol to examine outcomes related to healthcare access and quality for rural-dwelling pregnant white and AI individuals with OUD. Development of such a protocol is intended to lead to unique clinical insights about OUD and pregnancy in this study and to serve as a roadmap for future related work within the context of substance use disorders and pregnancy.

## Methods/design

### Step 1: Metric development

Metrics were developed within the context of our study population—three rural Utah counties with predominantly white and/or AI populations. We followed a process of literature review, guideline review, and stakeholder engagement similar to other pregnancy-related research [37]. We then identified specific knowledge gaps resulting from lack of direct observation to generate testable hypotheses for these knowledge gaps and develop metrics to test each hypothesis.

#### Review of literature and evidence-based guidelines

USP studies have evaluated access to mental health care, primary care, and OUD treatment; disparities in care; and quality of care for several primary care- and prenatal care-based complaints [18–20, 22–26, 35, 36, 38–50]. We therefore focused our review on these disciplines, seeking to adapt successful USP practices and protocols in other disciplines to the study of OUD in pregnancy.

Our review used a targeted, selective strategy to identify relevant USP studies through Medline and Google Scholar, including the search terms “audit study,” “simulated patient,” “standardized patient,” “secret shopper,” and “mystery shopper.” We associated these terms with applicable types of outpatient care, including “primary care,” “mental health,” “opioids,” “prenatal care,” and “obstetric care.” We then reviewed methodologies of identified studies. We found many applicable telephone-based approaches assessing access to care [19–23, 25–28, 38, 41] but relatively scant literature assessing quality of care through face-to-face visits [18, 35, 47, 51].

We followed the Donabedian model of structure, process, and outcome measures to derive our evaluation of quality [52, 53]. To date, few quality measures for OUD care in pregnancy have been defined [54, 55]; therefore, we reviewed existing recommendations for quality assessment, as well as current evidence-based guidelines for screening, risk factor probing, and treatment and management during an initial encounter to create a list of standards for comparison and measurement.

Members of the research team with expertise in obstetric and addiction care led a review of prenatal care guidelines [56] and screening recommendations for unhealthy drug use and prescription opioid misuse, including statements from the US Preventive Services Task Force and National Institute on Drug Abuse [57–61]. We focused on guidelines specific to OUD in pregnancy, including appropriate assessment of OUD severity, physical examination, and diagnostic workup [57, 62, 63]. Next, we reviewed screening guidelines for risk factors associated with adverse outcomes, including depression, anxiety, intimate partner violence, and other relevant factors [64–70]. We then reviewed guidelines for management of OUD in pregnancy [56, 71, 72]. Finally, we summarized these findings by category to represent characteristics of our target study population—a 22-year old pregnant female with OUD and prior intravenous drug use. See Additional file 1: Table S1.

Through expert-led team discussion, we determined that guidelines and prior literature emphasize the potential impact of a provider’s ability to assess OUD illness severity, comorbidities, risk factors for adverse outcomes, and life circumstances that might disrupt safe, successful treatment. We also concluded that how providers counsel patients about adherence, follow-up, and management would likely impact retention and treatment effectiveness. This iterative discussion process enabled us to prioritize measurement of relevant guidelines in our target study population.

We separately reviewed the literature for validated approaches to developing and embedding contextual factors (i.e., patient life circumstances affecting care) within USP profiles and identified applicable contextual factor domains for our evaluation [73]. We identified the Rochester Communication Rating Scale and Kalamazoo Essential Elements Communication Checklist for clinical communication, as well as several published articles and relating to stigmatizing language toward individuals with substance use disorders [74–79], to adapt measures of patient-provider communication and stigmatizing language.

#### Stakeholder engagement and expert consultation

We partnered with a network of federally qualified health centers within our rural study area with knowledge and cultural competency in caring for AI populations. Our purpose was to build study-specific context for evaluating knowledge gaps established through literature review. We specifically solicited input about enrollment and patient care processes; medical complaints/diagnoses commonly seen in primary, prenatal, and substance use care; cultural considerations for specific populations (e.g., AI patients); and areas of interest for quality improvement within the health center network. This mutually beneficial evaluation strategy engendered better cultural adaptation and alignment with local clinical priorities [51, 80]. For example, through our stakeholder discussions, we identified screening for alcohol use, unhealthy drug use, and mental illness as potential areas for concern. Measuring screening adherence therefore became an important area of evaluation for both our research team and the health center network.

We convened regular meetings with administrative and clinical leadership, identified a “confederate” (i.e., a clinical partner known to the research team but *not* to other clinical staff) at each clinical site to facilitate navigation of USPs within the clinical environment, and established a set of mutually beneficial evaluation priorities (e.g., quality improvement for substance use disorders). The confederate’s role is critical when routine processes for real patients, such as providing prior medical records, certain forms of identification, and certain diagnostic tests, create potential barriers for USPs. As an example, a real patient who is pregnant routinely provides a urine sample to confirm pregnancy during the first visit, but this creates a potential barrier for a USP reporting pregnancy (but not actually being pregnant). A confederate familiar with local processes can provide input and alternative solutions for these types of barriers.

In addition to stakeholder engagement, we consulted national experts in USP methodology. We specifically queried these experts about highly nuanced study aspects that must account for situational variables, such as clinic organization, scheduling procedures, healthcare information technology configuration, and USP recruitment and training. Expert input was iterative and incorporated into the protocol development at all stages.

#### Metric selection

**Fig. 1:**
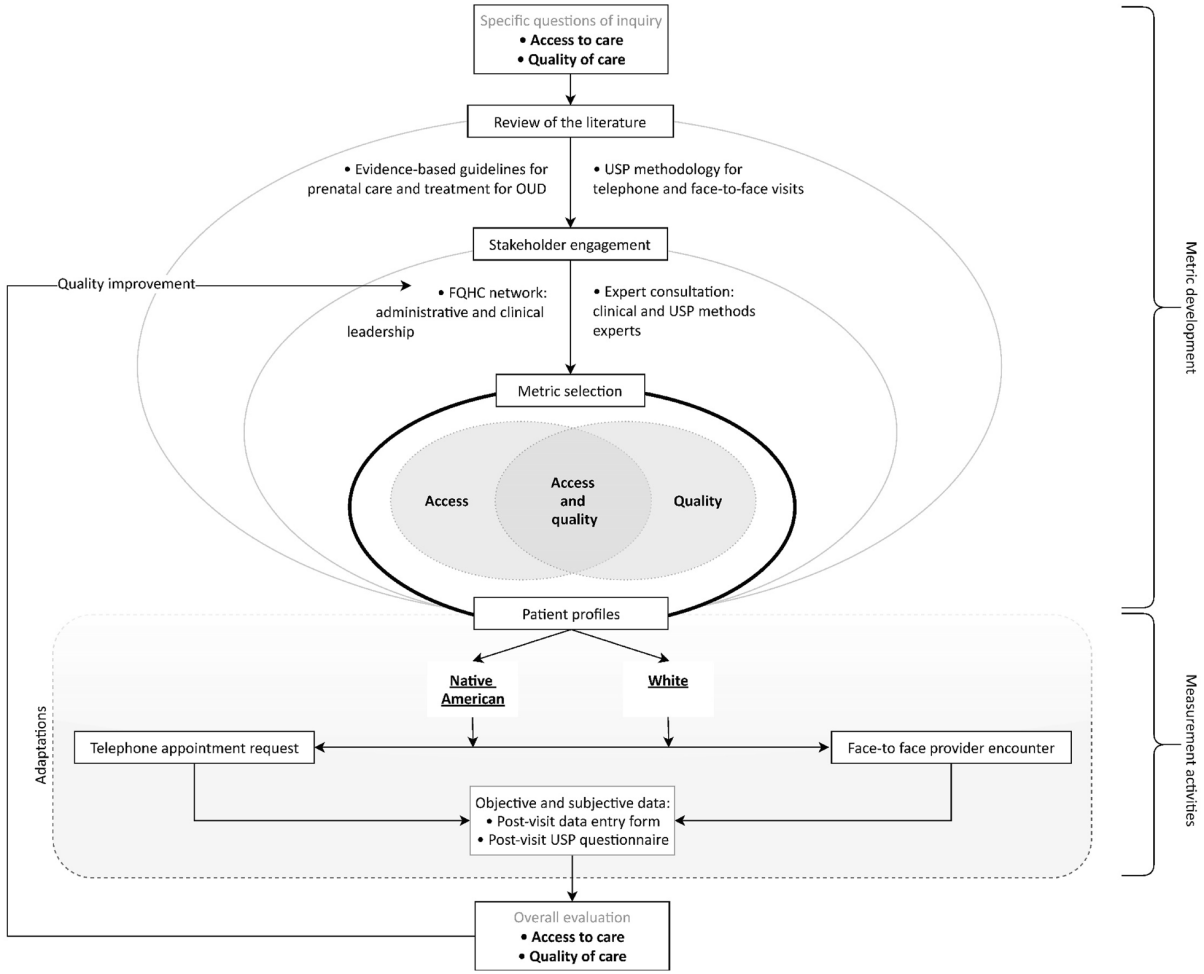
Process map for development of a USP methodology for pregnant white and American Indian individuals in rural areas

Selection of metrics was a critical step in adapting the USP methodology to our study, as study conclusions would be based in metric construct validity. Our objective was to broadly assess care from a patient’s initial telephone appointment request through completion of the initial provider visit. Metrics were selected to test six hypotheses about potential gaps in access and quality: (1) OUD knowledge among clinic staff is poor and creates a barrier to care; (2) access to OUD care for pregnant individuals in rural settings is limited by the availability of qualified providers and successful identification of those providers; (3) appropriate completion of evidence-based screening, symptom assessment, and management protocols are lacking; (4) risk factor screening/probing is not appropriately or reliably completed; (5) stigma is present; and (6) disparities in one or more of these five metrics exists between whites and AIs. See Fig. 1.

All metrics were established through team discussion and consensus following literature review and input from clinical stakeholders and experts. Because pregnant individuals with OUD face time-sensitive risks such as overdose or fetal harms, we determined appointment availability and wait times to be appropriate access metrics in this context [22, 25, 26, 38]. Additionally, because prenatal services and OUD treatment are often unavailable at a single site and many sites may have limited knowledge about OUD treatment, we sought to further examine access generated through clinic referrals. We developed a two-tiered calling protocol with uniform access metrics (familiarity/knowledge of OUD, treatment availability, willingness to treat, appointment wait time) for both primary and referral sites in our study sample.

We determined that testing hypotheses related to OUD care quality in pregnancy would largely require interaction with a clinic and provider during an initial patient visit. Because several aspects of care quality are difficult to assess in a single visit, we prioritized screening and management metrics most critical to an *initial* encounter, such as provision of naloxone and identification of other central nervous system depressant use (e.g., benzodiazepines). Metrics related to contextual factors followed an established pattern of disclosing a clue or “red flag” about a potential problem or risk (e.g., “I’m not taking my medicine like I normally do”), then assessing whether the provider inquired or probed to understand the underlying contextual factor (pill-sharing with a friend) [73].

**Fig. 2:**
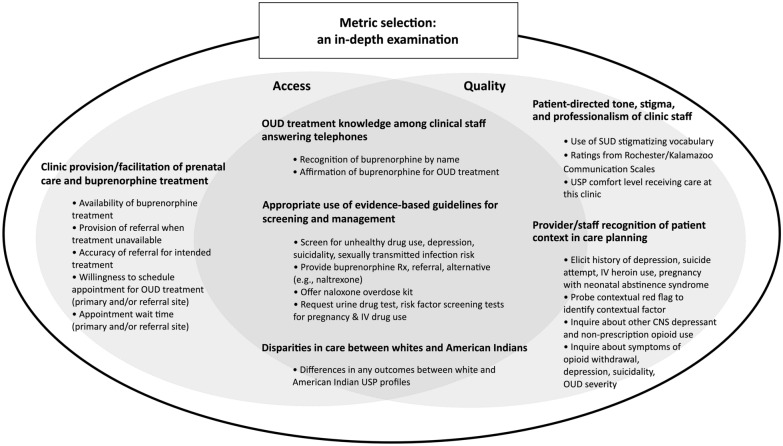
Metrics targeting specific outcomes to measure healthcare access and quality for pregnant white and American Indian individuals with OUD

In total, 18 metrics—for access, quality, or both—aligned with the six hypotheses tested (see Fig. 2).

### Step 2: Patient profiles

“Patient profiles” are the standardized patient characteristics assigned to each USP. Our objective was to create patient profiles representative of white and AI pregnant women with OUD residing in rural Utah that would capture the data required for each outcome metric. We sought input from several sources, including prior studies, the health center network, and others with lived experience in rural Utah communities, AI communities, or both (see Fig. 1). Additionally, we engaged a research assistant of Navajo descent familiar with local cultural practices and knowledgeable about common social determinants of health, insurance status, and other contextual factors within the Navajo community. Given that other research team members had extensive experience treating pregnant patients with OUD of all races/ethnicities who reside in the sampling area, the team’s collective experience allowed for a basic understanding of environmental and cultural considerations in creating the USP profiles.

We created two identical patient profiles that differed only by patient race (white or AI) and race-related context (e.g., name, place of previous residence), shown in Table 1. Callers would represent either the patient or the patient’s male partner for the white profile, but only the patient for the AI (Navajo) profile. The health center network provided additional insights about developing AI/non-AI profiles as well. For example, a Navajo individual is likely to relocate from reservation lands, while a white individual is likely to relocate from non-reservation lands. Similarly, Navajo communities tend to follow a matriarchal order, so while a male partner calling on behalf of the patient would be appropriate for a white couple, it would be highly unusual for a Navajo couple.

**Table 1:** Standardized Patient Profiles

	White/Caucasian	American Indian (Navajo)
Demographic information		
Name	Leah Lapinski	Sasha Tso
Birth date	12/04/1997	
Age	22	
Relationship	Boyfriend, 1 child (age 2)	
Employment	Not working/unemployed	
Insurance	Medicaid	
Personal information		
Address	[vacant property in nearest municipality to clinic] OR [unavailable; staying with friend, moving to apartment]	
Phone	[borrowing from a friend]	
Email	trailwanderer95@gmail.com	
Previous provider	“Dr. Patel”	
Chief complaint	New prenatal care	
Patient concerns	• Heroin relapse • Loss of child custody • Becoming suicidal again • Health/wellbeing of new baby • Financial stress/unemployment	
Background		
Recent relocation from	Denver, CO	Navajo Nation (Window Rock, AZ)
Medical history	Chronic neck pain • after motor vehicle accident ~ 7 years ago, treated with oxycodone for several years Opioid use disorder • arising from chronic oxycodone use, was aggressively tapered by previous provider, began using IV heroin • During pregnancy of first child, sought OUD treatment, started on Suboxone, child was hospitalized for 6 weeks with neonatal abstinence syndrome • Had one relapse with heroin for 2 months, has been stable on Suboxone without relapse for last 12 months Depression • Treated medically by PCP for last 4 years • 1 suicide attempt (oxycodone overdose) about 4 years ago	
Allergies	None	
Medications	Suboxone 8 mg/2 mg BID Zoloft 100 mg/day Prenatal vitamin	
Immunizations	“up to date”	
Set-up instructions (clinic visit only)		
Clothing	Elastic exercise pants, dingy t-shirt, coat/jacket, disheveled hair	
Equipment	Backpack, smart phone, snack (in backpack)	Backpack with attached dreamcatcher, smart phone, snack (in backpack)
Position	Sitting in chair (or on exam table if no chair available)	
Symptoms (clinic visit only)	• Occasional neck spasms/pain, 6/10, sometimes improves a little with Motrin • Depressed/anxious: worried about pregnancy, finances/unemployment • No suicidality	

After discussions and consensus among team members, we identified several patient profile characteristics to enhance the quality of our evaluation. First, we determined buprenorphine continuation, rather than initiation, would be more likely accepted by buprenorphine prescribers and capture the highest possible number willing to treat OUD in pregnancy. Second, in our literature review, we identified barriers and facilitators to seeking care, such as maternal concern for loss of child custody [81]; therefore, we incorporated these characteristics and other established risk factors to increase urgency of the request and evaluate whether these characteristics are recognized/addressed by clinical staff. Third, we limited the scope of extraneous medical issues to focus only on measures of interest. Only comorbidities that could potentially modify OUD and prenatal treatment, such as depression or other substance use, were included. A comprehensive description of USP profiles and script prompts are available in Additional file 1: Exhibit S1.

### Step 3: Encounter protocols

Pregnant individuals with OUD interface the healthcare system as new patients primarily through two encounters: scheduling an appointment and meeting with a provider during an initial visit. Because each may affect delivery of care, we designed two interrelated USP protocols to account for both. Together, these two protocols capture all metrics required for the six access and quality outcomes. The telephone appointment request was deemed as not human subjects research by the University of Utah IRB; the face-to-face visit was a quality improvement project and not subject to IRB review.

#### USP recruitment

We considered several factors in recruiting individuals as USPs for this study. We first sought individuals with experience in prior USP studies, as it comprises a unique skillset and individualized training. Without experienced USPs readily available, we identified candidate research assistants and began a formal training program with an academic expert in USP studies and a long track record in coaching and training USPs. While some USPs have training in acting, our academic expert discouraged recruitment of individuals with this background as acting in clinical encounters can become too embellished and present red flags to providers and staff. In contrast, phenotype—providing an accurate audiovisual representation of the intended profile—was very important. Thus, we recruited a) two USPs who were native to the study region who identified as American Indian females, and b) two USPs residing in Utah who identify as white.

#### Telephone appointment request

Since the first point of patient contact with a clinic provider is frequently by telephone, we determined to use a phone call protocol modeled after Tipirneni et al.’s evaluation of primary care access [22] to assess (a) familiarity with and knowledge of OUD and OUD treatment; (b) availability of prenatal care and OUD medication treatment; (c) willingness to provide prenatal and OUD care (i.e., schedule appointment); and (d) appointment wait times in calendar days. We then developed an algorithm to determine the availability of prenatal care, followed by availability of OUD treatment, and finally, the provision and quality of referral if either prenatal care or OUD treatment is not available. Additionally, we developed a coding algorithm for call recordings to identify whether clinical staff use stigmatizing language/tone and recognize/probe warning signs that the patient’s circumstances create additional risk for an adverse outcome (i.e. contextual and medical red flags) [73]. See Additional file 1: Figure S1.

Previous telephone-based USP studies have shown that clinical staff make patient inquiries that create barriers for USPs to obtain needed data. We therefore developed prospective answers to those inquiries as “work-arounds” to obtain the information of interest. Because several of our study metrics assess clinic knowledge about OUD treatment and recognition of risk factors, we separately developed an algorithm for the timing of information disclosure. For example, whether and when to reveal prior heroin use or current pill-sharing practices could affect our ability to determine whether clinics inquire about that information or how they use initial information presented. The disclosure protocol and USP training guide listing all work-arounds are available in Additional file 1: Table S2.

We designed the protocol so each clinic would be contacted twice (once for each patient profile); therefore, we planned a 3-week “wash-out” period between calls to minimize priming/suspicion when the same staff member answers both calls. This brief time interval between calls was balanced against potential bias introduced by an excessive time lag. It is important to note that we ensured USP appointment requests would not displace appointments for real patients, by either canceling or declining to accept offered appointments.

We selected Google Voice to place calls, which allows for use of an area code representative of the sampled geographic area. To avoid callbacks, we developed a work-around that the phone belonged to a friend and the USP would not have access to it later. To assure clinical staff would recognize the USP as AI when indicated, four discrete clues were embedded into the profile and additional clues voluntarily disclosed *ad lib* by the USP were built into the AI USP script (e.g., “…when I was getting care at the Indian Health Service…”). While USPs calling for the white profile did not state they were white when asked, we used names that are characteristically white, and only white individuals with local accents made the calls.

#### Face-to-face visit

Following our study objectives, we chose a face-to-face provider visit to observe clinician behavior, assess quality of care, and measure the ability or willingness of the provider to continue care and/or refer when OUD is disclosed for the first time during an initial encounter. An algorithm for face-to-face visits is presented in Additional file 1: Figure S1. Input from partners in the health center network was needed to develop new work-arounds, such as for invasive laboratory tests and procedures, in developing the face-to-face approach. For example, partners recognized that patients in the health center network often have limited clinic time because their transportation depends on another person. Using time constraints due to the schedule of the USP’s transportation assistance was therefore a natural barrier to same-day laboratory work.

### Step 4: Sampling and analysis

Clinical sites delivering primary care and obstetric care in three rural counties were identified through a commercially available database of provider listings by specialty (IQVIA), combined with verification of database information through internet search and consultation with stakeholder confederates. A total of 18 clinical sites meeting criteria were identified. See Additional file 1: Table S3.

We developed a coding protocol for telephone appointment requests and face-to-face visits using input from team members and prior USP studies [82]. Because outcome measures included both objective and subjective data, objective findings were captured through review of audio recordings using a post-visit data entry form, and subjective data were captured through a post-visit questionnaire completed by each USP immediately following the encounter. See Tables 2 and 3. We selected REDCap to tabulate and export data for analysis [83, 84] and later manual coding, review, and descriptive statistics. The overall objective in creating our analysis plan was to measure how often each objective performance metric is met and describe the degree of alignment between provider performance and societal guidelines for subjective performance metrics.

**Table 2:** Post-encounter data collection for *telephone appointment request*

TELEPHONE APPOINTMENT REQUEST: coding team post-visit data entry form	
1. Clinic name, location	2. Clinic staff member answering call (i.e. scheduler, nurse)
*OUD treatment information*	
1. Had the person who answered the phone heard of Suboxone before? ↳ [IF YES] level of familiarity 2. Asked the specific reason why patient needed to take the Suboxone?	3. OUD treatment available at this site? ↳ [IF NO] Referral offered? Contact info provided? 4. Was an appointment offered for OUD treatment? ↳ [IF YES] Provider and date_________________ ↳ [IF NO] Reason appointment not offered ↳ [IF NO] Referral offered? Contact info provided?
*History and context*	
1. Asked about duration of pregnancy or about patient's last menstrual period? 2. Asked for information about patient's previous care/provider?	3. Inquired about patient's own understanding of their medical conditions? 4. Asked about patient's insurance coverage? 5. Asked where patient is moving from?
*Risk stratification/triage*	
1. Asked whether patient had a current supply of buprenorphine/Suboxone? 2. Probed when patient disclosed aberrant dosing practice? 3. Asked about patient's mental health risk factors?	4. Allowed patient to speak with clinical person (i.e. nurse) when scheduler's knowledge about their issues was limited? 5. Any clinic staff asked if patient had any other concerns?
*Prenatal treatment information*	
1. Was a prenatal appointment offered? ↳ [IF YES] Provider and date_________________	↳ [IF NO] Reason appointment not offered ↳ [IF NO] Referral offered? Contact info provided?
*Other appointment information*	
1. Was patient's contact information requested?	
*Medical disclosures*	
1. Asked if patient takes any medications/other medications? ↳ [IF YES] Asked about depression after Zoloft disclosed? ↳ [IF YES] Asked about suicidality after depression disclosed?	2. Inquired where current supply of medications were prescribed from?
*Encounter flow*	
1. Was the patient cut off while explaining their situation, concerns, and requests?	2. Were any questions avoided from being answered?
*Word choice*	
1. Were any of the following words used by the health care organization on the call? [list of terms]	
*Call duration information*	
1. Total duration of call 2. Time speaking with scheduler 3. Time waiting/on hold	4. Number of times placed on hold 5. Other time

TELEPHONE APPOINTMENT REQUEST: Post-visit questionnaire	
1. Call attempt # __________________ 2. White or American Indian profile? 3. Appointment offered?	4. If no MOUD treatment offered: were you referred to another provider? 5. Based on your phone encounter, how comfortable would you feel receiving your care at this clinic?
*I felt my scheduler/nurse…[level of agreement]*	
1. Greeted me warmly 2. Let me explain my problem without interruption 3. Did not seem distracted 4. Asked me if I had any questions 5. Used words that show care and concern throughout the call 6. Used a tone and pace that show care and concern 7. Summarized my information and gave me the opportunity to correct or add information 8. Transitioned effectively to additional questions when gathering information 9. Responded explicitly to my statements about ideas and feelings regarding my questions and concerns 10. Other comments (free text):________________

**Table 3:** Post-encounter data collection for *face-to-face provider encounter*

INITIAL PROVIDER VISIT: coding team post-visit data entry form	
1. Clinic name, location, date, time	2. Provider name, gender, specialty, degree
*Check-in and triage information*	
1. Asked about records from previous provider? 2. Said services offered to me would be affected by my insurance status? 3. Asked about duration of pregnancy or about patient's last menstrual period? 4. Urine pregnancy test requested? 5. Screened for unhealthy drug use? ↳ [IF YES] Probed about IV drug use in past/last use?	↳ [IF YES] Screening was otherwise completed as directed by USPSTF? ↳ [IF YES] Positive screen was communicated to provider? 6. Screened for depression? ↳ [IF YES] Specific screening tool used (and which)? ↳ [IF YES] Tool used appropriately/as indicated? ↳ [IF YES] Asked about recent or current suicidality? ↳ [IF YES] Positive screen was communicated to provider?
*Provider encounter*	
1. Asked about current medications and doses? ↳ [IF YES] Inquired where current supply of medications were prescribed from? 2. Identified/discussed patient history of depression? ↳ [IF YES] Specific screening tool used (and which)? ↳ [IF YES] Tool used appropriately/as indicated? ↳ [IF YES] Asked about recent or current suicidality? 3. Identified/discussed patient history of IV heroin use? 4. Identified/discussed patient history of pregnancy complicated by neonatal abstinence syndrome? ↳ [IF YES] Addressed concerns about NAS in current pregnancy 5. Screened for sexually transmitted infection risk?	6. Asked for information about patient's previous care/provider? 7. Inquired about patient's own understanding of their medical conditions? 8. Probed on contextual red flag (“I am not taking my medication the way I usually do”)? ↳ [IF YES] discussed concern and/or amended plan when contextual factor (pill-sharing) was disclosed? 9. Asked about OUD severity? 10. Asked about symptoms of opioid withdrawal? 11. Asked about recent or concurrent use of other CNS depressants or illicit substances? 12. Asked if patient had any other concerns?
*Provider management*	
1. Offered naloxone overdose kit? 2. Requested urine drug testing? 3. Screened for sexually transmitted infections? 4. Screened for infections in people who inject drugs?	5. Offered/prescribed medication treatment for OUD? ↳ [IF NO] Reason treatment not offered______________ ↳ [IF NO] Offered appropriate referrals?
*Word choice*	
1. Were any of the following words used by the health care organization on the visit? [choose from list of terms]	
*Encounter flow*	
1. Was the patient cut off while explaining their situation, concerns, and requests?	2. Were any questions avoided from being answered?
*Appointment follow-up*	
1. Return appointment requested? ↳ [IF YES] Time interval or date or return appointment________	↳ [IF NO] Reason return appointment not offered____________
*Appointment duration information*	
1. Total duration of appointment (check-in to check-out) 2. Time in waiting room 3. Time in exam room waiting for provider	4. Time in exam room with provider 5. Other time

INITIAL PROVIDER VISIT: USP post-visit questionnaire	
1. White or American Indian profile?	2. Based on your phone encounter, how comfortable would you feel receiving your care at this clinic?
*I felt my provider…[level of agreement]*	
1. Greeted me warmly 2. Used tone, pace, eye contact, and posture that show care and concern 3. Asked about all of my concerns early in the interview (usually by asking 'anything else") 4. Made me feel I could tell him/her anything, even something personal 5. Let me explain my problem without interruption 6. Allowed me to tell my story in my own words 7. Did not seem distracted 8. First asked about my general concerns, then asked about specific details 9. Transitioned effectively to additional questions when gathering information	10. Asked about life events, circumstances, other people that might affect health 11. Made an effort to understand my feelings and emotions 12. Summed up and made sure they understood what I said (without putting words in my mouth) 13. Gave me the opportunity to correct or add information 14. Asked me if I had any questions 15. Responded explicitly to my statements about ideas and feelings regarding my questions and concerns 16. Checked to see if I was willing and able to follow through with the treatment plan 17. Summarized/asked me to summarize plans until next visit and/or clarified follow-up or contact arrangements 18. Additional comments (free text):_____________________

With the exception of disparities between white and AI USP encounters, all other metrics in our study are descriptive in nature. Therefore, for qualitative aspects of our study, our sample size of all 18 sites in the sample area is sufficient to achieve thematic saturation [85]. Disparities in outcomes are tested using two sample, two-tailed *t-*tests. Determining a sample size to appropriately power our study and identify disparities (if present) is difficult for two reasons. First, unlike most clinical trials, the variance of primary and secondary outcomes is rarely known (and is not known for outcomes in this study). Second, defining a clinically meaningful difference in many outcomes (e.g., appointment wait times) can be subjective. Because relatively few clinical sites exist within the rural region of our study, we chose to include all 18 sites (saturated sample). However, by making a few pragmatic assumptions, the reasonableness of the sample size can be estimated. If, for example, a difference of 7 days were present between the two groups with a mean wait time for the reference group and a standard deviation of 0–14 days and 7 days, respectively, 16 independently sampled sites would be sufficient for a power level of 0.8. Similarly, if a relative disparity of 20 percent—one likely to be clinically meaningful—were present for referral to treatment (e.g., 40 percent for AI compared to 50 percent for white), with a standard deviation of 16 percent, 11 sites would need to be independently sampled for each profile to achieve a power level of 0.8. Our sample size exceeds the minimum requirement based on these assumptions.

### Step 5: Pilot testing, process evaluation, and adaptation

The telephone appointment request protocol was tested through six pilot calls made to clinics in or near the sampling area. Callers received two separate training sessions facilitated by USP experts on the research team. These calls were audited and reviewed by investigators of the research to team to establish protocol face validity. Additionally, we used the calls to align with regional clinical practices and correct logic in post-visit data entry forms. For example, USPs were sometimes transferred immediately from a front desk scheduler to a medical assistant or nurse without an opportunity to request an appointment. These adjustments were made after each call until intended metrics could be reliably assessed.

A second purpose of the pilot call period was to verify USP fidelity. Fidelity checks assure protocol delivery across different USPs is consistent and reliable [31]. Fidelity checks in this study are especially important to ensure reliability between male and female USPs. As a matter of practice, we perform the same fidelity check on the entire study sample after data collection to ensure a consistent result. Our fidelity check used (1) a qualitative approach to compare USP performance to the written protocol by auditing the calls and providing feedback, and (2) a quantitative approach to assess for statistical differences between responses of randomly assigned clinic sites for each USP. A summary of process evaluations and outcomes for our method development is presented in Table 4. As shown, adaptation of the USP method to this clinical context relied on expertise specific to the patient population and medical condition, as well as standard processes to assure quality and fidelity of data collection.

**Table 4:** USP protocol evaluation processes and outcomes for pregnant white and American Indian individuals in rural areas

Step in protocol development	Process(es)	Outcome
Metric selection	Literature review	18 metrics aligned with prior literature, evidence-based guidelines, and stakeholder input developed to test 6 hypotheses relevant to treatment of OUD in pregnancy
	Expert consultation	
	Stakeholder engagement	
Profile development	Adaptation from prior studies	2 regionally, culturally representative profiles created
	Expert consultation	
	Stakeholder engagement	
Pilot calls	USP training	Callers familiarized with protocol dialogue and refinements made to training guide to reflect sampling context
	Metric capture/Refinement of post-visit data entry forms	Reliable metric capture obtained
	Fidelity checks	Qualitative fidelity observed across USPs

The novel coronavirus SARS-CoV-2 (COVID-19) pandemic emerged during the development of our USP protocol. We accounted for these restrictions in our telephone appointment requests by (1) defining COVID-19 as a distinct and separate barrier to care when requesting an appointment and asking what clinics would do “under normal operating conditions” if care was refused due to COVID-19; and (2) allowing for virtual visits to be scheduled instead of face-to-face visits if necessary. For face-to-face encounters, we continue to work with our stakeholders to assure safe timing.

## Discussion

We have described the development of a USP methodology to define and evaluate healthcare access and quality for white and AI pregnant individuals with OUD residing in rural communities. This novel application of the USP approach will allow for improved understanding about access, quality, and potential disparities in OUD treatment not previously identified or reported, as it has shown elsewhere [86].

With fewer physicians per capita and less than half of rural counties offering hospital-based obstetric services in the US [87–89], access to maternal healthcare and substance use care in rural areas is limited in comparison to non-rural areas. Behavioral health services for substance use disorders are also not widely available in rural settings—the lack of which has been associated with deleterious neonatal outcomes [88, 90, 91]. Evaluating access to care for this population has been challenging because of ill-defined measures and lack of data; however, the USP approach enables assessment of access not previously established for this population and may identify interventions to improve access to care.

While quality of care for pregnant individuals with OUD has not been well defined, this approach allows for comparison of clinic and provider performance to evidence-based guidelines that cannot be accurately or fully measured with administrative data. Certified buprenorphine prescribers represent less than 10 percent of rural providers and are more likely to practice primary care than specialty care in comparison to non-rural areas [88, 92]. Understanding the quality of care delivered among this comparatively less specialized workforce may identify opportunities for quality improvement interventions that will increase care quality and reduce harms.

Further, the USP design can be used longitudinally to assess performance before and after quality improvement interventions are implemented to determine their effectiveness in achieving desired outcomes [51]. Our approach intentionally leveraged a partnership with clinical stakeholders to not only identify access deficiencies and quality but also to develop, implement, and evaluate interventions [18, 51]. Observations and input from clinical staff were helpful in both refining hypotheses to be tested and in aligning research queries with actionable changes to improve care.

Our study is unique in its application of an established approach for an understudied population and also because it assesses healthcare access and quality across an episode of care. The steps of assessment—beginning with calls to local primary care providers, and ending with completion of an initial patient encounter—provide insights, such as clinic familiarity with treatment, process barriers, appointment wait times, referral adequacy, and signals of quality invisible in administrative data. To date, USP studies have traditionally focused on only one dimension of care (e.g., wait times) without examining clinic/provider behavior for subsequent steps patients would be expected to take in their own care. Integrated data across a continuum of patient care from initial contact to completion of an appointment provides broader insights into where and when barriers to care present, and whether and how treatment varies by clinic, region, or race. Both protocols were also constructed for simultaneous assessment of multiple outcomes related to access, quality, and stigma/bias. The richness of the data can therefore establish relationships between measures of access, quality, and stigma using a single observational data collection.

There are also several limitations to our approach. First, our study is cross-sectional and provides information at only one point in time. However, the method allows for repetitive evaluation over time. Second, the use of multiple USPs may create unintended biases in data collection. We account for these biases by comparing primary and secondary data outcomes across USPs to assure no correlation is present. Third, the unit of analysis in this study is a clinical site, and in sites with more than one prenatal provider, data for face-to-face visits only represent one provider at that site, rather than the clinic as a whole. Further study, stakeholder collaboration, and development of this protocol will allow for saturated sampling of all providers in sampled clinics, as has been done in USP studies elsewhere [47]. Fourth, our protocol was developed in a 3-county setting in rural Utah with input from a local AI research team member and health center network that are both specific to one AI tribe, which may limit generalizability. However, many aspects of the study setting are similar to rural and AI areas throughout the US. Fifth, environmental conditions, such as COVID-19, may have biased results, and additional resources are required to validate the methodology under non-pandemic circumstances.

### Next steps and conclusion

Following completion of data collection and analysis, findings will be reported back to clinical partners to begin a process of quality improvement. Subsequent re-evaluation will then be used to assess changes in quality over time. We will further use this approach to adapt the USP protocol for evaluation in other communities.

OUD in pregnancy remains a critical concern in the US, especially among vulnerable populations residing in rural communities. We have described a novel USP protocol to assess healthcare access and quality for pregnant, rural-dwelling white and AI individuals that cannot be measured through administrative data. While much work remains to improve care for OUD in pregnancy and reduce disparities, this protocol represents a step toward gaining new insights and may serve as a roadmap for future healthcare access and quality research within the context of substance use disorders and pregnancy.

## Supplementary Information

**Additional file 1.** (1) Summary of evidence-based guidelines for opioid use disorder in pregnancy; (2) unannounced standardized patient training guide; (3) unannounced standardized patient disclosure protocol; (4) summary of study sample; and (5) unannounced standardized patient encounter algorithms.

## Data Availability

Not applicable.

## Abbreviations

AI: American Indian/Native American
COVID-19: SARS-CoV-2 novel coronavirus disease
IV: Intravenous
OUD: Opioid use disorder
USP: Unannounced standardized patient
