# Supplementary material for: Development of an unannounced standardized patient protocol to evaluate opioid use disorder treatment in pregnancy for American Indian and rural communities
Source: Addict Sci Clin Pract. 2021 Jun 25;16:40. doi: 10.1186/s13722-021-00246-6 (PMC8229269; doi:10.1186/s13722-021-00246-6)

**Development of an unannounced standardized patient methodology to evaluate**

**opioid use disorder treatment in pregnancy for American Indian and rural communities**

**Appendix**

Table of Contents

[**Table S1.** Evidence-based guidelines for the care of a 22-year old pregnant woman with opioid use disorder and history of IV drug use. 4](#_Toc53699843)

[**Exhibit S1**. Standardized Patient Training Guide 5](#_Toc53699844)

[1. Background/Objectives 6](#_Toc53699845)

[Background 6](#_Toc53699846)

[Study Design 6](#_Toc53699847)

[Objectives 6](#_Toc53699848)

[2. Standardized Patient Call Method 6](#_Toc53699849)

[Your job as a caller: 7](#_Toc53699850)

[Terminology 8](#_Toc53699851)

[Using Google Voice to Call the Clinics: 8](#_Toc53699852)

[3. Profiles of Callers 9](#_Toc53699853)

[Medical Condition Profile(s)*: 9](#_Toc53699854)

[Insurance Profile(s): 9](#_Toc53699855)

[4. Scripts/Flow Chart 10](#_Toc53699856)

[*Must be stated during call:* 10](#_Toc53699857)

[*If asked:* 10](#_Toc53699858)

[*Flow Chart:* 12](#_Toc53699859)

[5. Work-Arounds 12](#_Toc53699860)

[Not accepting new patients due to COVID-19 12](#_Toc53699861)

[Not doing face-to-face visits due to COVID-19 13](#_Toc53699862)

[Medicaid 13](#_Toc53699863)

[Insurance 13](#_Toc53699864)

[Other Work-Arounds 13](#_Toc53699865)

[6. Data Collection 15](#_Toc53699866)

[Post-visit questionnaire 15](#_Toc53699867)

[Coding of recorded audio 16](#_Toc53699868)

[Data entry best practices 16](#_Toc53699869)

[**Table S2.** Disclosure protocol 17](#_Toc53699870)

[**Table S3.** Sampled clinics by type 17](#_Toc53699871)

[**Figure S1.** Telephone Appointment Request and Face-to-face Visit Algorithms for Unannounced Standardized Patients with Pregnancy and Opioid Use Disorder. 18](#_Toc53699872)

# Table S1. Evidence-based guidelines for the care of a 22-year old pregnant woman with opioid use disorder and history of IV drug use.

| **Guideline** | **Category** | **Measurable instruments, tests, or inquiries** |
| --- | --- | --- |
| Screening | Unhealthy drug use | 4Ps  Alcohol, Smoking, Substance Involvement Screening Test (ASSIST)  Audio Computer-Assisted Self-Interview (ACASI) ASSIST  CRAFFT  National Institute on Drug Abuse (NIDA) Quick Screen |
|  | Depression | Edinburg Postpartum Depression Scale (EDPS)  Patient Health Questionnaire 2-item (PHQ-2)  Patient Health Questionnaire 9-item (PHQ-9) |
|  | Suicide Risk | Columbia-Suicide Severity Rating Scale (C-SSRS)  Suicide Assessment Five-Step Evaluation and Triage (SAFE-T) |
|  | People who inject drugs | Hepatitis B  Hepatitis C  HIV  Latent tuberculosis |
|  | Sexually transmitted infections | Chlamydia (and gonorrhea when risk factors present)  Hepatitis B  HIV  Syphilis |
|  | Pregnancy | Antibody screen  Baseline urine protein  Blood type and D (Rh) Type  Complete blood count  Fetal ultrasound  Gestational history and related complications |
| OUD Severity | Review of Symptoms | DSM-V Criteria  TIP 63 (SAMHSA) |
|  | Risk-taking behaviors | Diversion  Non-prescription opioid use  IV drug use  Other CNS depressant use |
|  | Physical Exam | TIP 63 (SAMHSA) |
| OUD Management | Medication treatment | Buprenorphine monoproduct  Buprenorphine and naloxone  Methadone  Natrexone |
|  | Overdose | Naloxone overdose nasal spray or injector |
|  | Withdrawal symptoms | *Pharmacologic*  Clonidine  Acetaminophen  Ibuprofen/NSAIDs  Dimenhydrinate  Hydroxyzine  Ondansetron  *Non-pharmacologic*  Hot baths/showers  Resting  Walking |
|  | Monitoring and follow-up | Urine drug testing  Appropriate visit frequency |

# Exhibit S1. Standardized Patient Training Guide


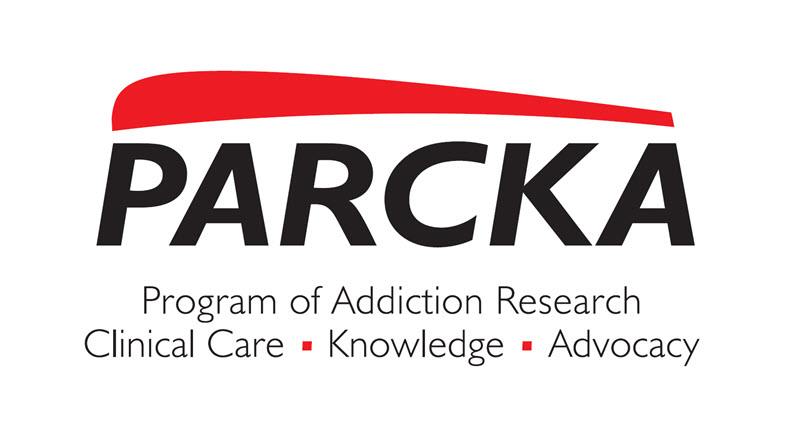


**Standardized Patient Call Training Guide**

***Evaluation for Pregnant Women with Opioid Use Disorder in Rural Utah***

*Adapted from:*

*Simulated Patient Call Training Guide for the evaluation of primary care access before and after Medicaid expansion in Michigan—Renuka Tipirneni, MD, MSc, 2014*

## Background/Objectives

### Background

Opioid-related overdose is a major public health problem in Utah, which has the second highest rate of opioid-related death among western states. Opioid use in pregnancy is of particular concern; across the US, diagnosis of opioid use disorder (OUD) among pregnant women has quadrupled in the last decade, and in Utah drug-induced death is now the leading cause of pregnancy-associated mortality. Moreover, most pregnant women do not receive evidenced-based medication-assisted treatment for OUD (M-OUD). Whether these women have adequate access to prenatal care and M-OUD is not well understood.

Therefore, there is a critical need to evaluate access to comprehensive prenatal care and M-OUD for pregnant women with OUD in Utah. The goal of this study is to assess *new patient appointment access* for pregnant women with OUD in Carbon, Emery, and San Juan Counties in Utah. This study is part of a broader needs assessment commissioned by the Utah Division of Substance Abuse and Mental Health related to pregnant women with OUD in rural Utah, where the extent of the opioid crisis among this high-risk population is not currently known.

### Study Design

Using an unannounced standardized patient approach (a.k.a. audit or “secret shopper” study), we will contact all community practices providing prenatal and obstetric care in Carbon, Emery, and San Juan Counties to determine a) whether the clinic will accept the patient given the presenting complaint and what the expected wait time for a new appointment will be; b) the effect of introducing a contextual factor (loss of access to a prescribing physician leading to dose-sharing with a friend) on access measures; c) the manner of treatment of the patient in terms of stigmatizing language, tone, accommodation, and other patient-directed attitudes; and d) referral patterns and practices among clinics unable or unwilling to treat the conditions presented. Two standardized patient profiles will be used:

1. A white/Caucasian woman seeking prenatal care in setting of opioid use disorder treated with buprenorphine; and
2. An American Indian/Native American woman seeking prenatal care in setting of opioid use disorder treated with buprenorphine

Access will be measured by a) whether the practice will accept the patient; b) whether alternatives are offered if the patient cannot be accepted; and c) the appointment wait time in calendar days. Additionally, standardized patients will complete a brief questionnaire after each call, and audio recordings of the phone calls will be coded to evaluate evidence of stigmatizing language and bias, appropriate probing and triaging of red flags for patient risk, and variability in clinic scheduling and enrollment practices. All metrics will be compared across both patient profiles.

### Objectives

1. Evaluate and compare new patient experience and access to prenatal care in Carbon, Emery, and San Juan Counties for pregnant women with OUD.
2. Evaluate and compare experience and access to prenatal care and M-OUD between white/Caucasian and American Indian women.
3. Validate a novel unannounced standardized patient approach to conduct a broader evaluation of access to prenatal care among women with OUD in Utah and the rural US.

## Standardized Patient Call Method

You will be trained to pose as a 22-year old female (or her boyfriend) and you are new to the area. You are approximately 2 months pregnant and are calling to request a new patient prenatal care appointment. Calls will be made to each clinic/practice twice (once for each patient profile), with calls to the same clinic separated by 2-4 weeks.

- By *prenatal care in the setting of OUD*, we mean the patient has been diagnosed with opioid use disorder (OUD) in the past and has been prescribed buprenorphine with successful management prior to this pregnancy. You are now is seeking prenatal care and hope your buprenorphine will be able to be continued by your new prenatal provider or someone the provider would recommend.
- Other relevant history:
  - You recently moved to the area
    - From Denver area (white/Caucasian profile)
    - From Window Rock, Arizona (American Indian/Native American profile)
  - You first became dependent on opioids through a prescription after a car accident several years ago that resulted in chronic neck pain. When your doctors stopped your prescriptions, you began to use heroin, and you were on heroin when you became pregnant with your last (and only) child. You transitioned to buprenorphine during pregnancy, but your baby was hospitalized for 1 month after birth and treated for neonatal abstinence syndrome. You had a brief heroin relapse 1 year ago but got back on buprenorphine and have been successfully treated for the last 8 months.
  - Child protective services (a.k.a. DCFS in UT, Division of Child Welfare in CO, Department of Child Safety in AZ) was involved in the birth of your first child. You did not lose custody, but you know that if you relapse, custody of your children could be in jeopardy.
  - You recently lost access to your previous buprenorphine prescriber (Dr. Patel), so for the last several weeks you have been pill-sharing with a friend so you don’t relapse.
  - You have depression and take Zoloft. You had one suicide attempt when on taking opioid pills for pain after your accident (intentional overdose).

For each call, research assistants will be randomly assigned. Calls to the same clinical site/facility for different patient profiles will be separated by 2-4 weeks to avoid recognition of calls where similar characteristics may be recognized. Actual making of appointments will be avoided/canceled at the end of the call so that appointment slots are not taken from real patients seeking care.

Calls will be made remotely by University of Utah research staff using Google Voice to simulate patient sham phone numbers. All call recordings and other data will be stored on a HIPPAA-compliant storage cloud requiring 2-factor authentication for access.

### Your job as a caller:

The most important piece of information we are trying to collect is whether or not the clinic will provide the treatment you are seeking and what resources are offered if the clinic cannot or will not provide treatment. If we wanted to just ask the question hypothetically, we wouldn’t need to use the deceptive practice of posing as patients. The reason we are going through the trouble of posing as patients is to get a REAL answer. In order to get a REAL answer, we have to get specific information (e.g., willingness to accept you as a patient, a specific appointment date, etc.). We will also be assessing the way the clinic addresses the caller’s concerns over the phone, the overall professionalism shown to the caller, and the ability of schedulers and trained medical staff to recognize red flags that the patient may be at risk for an adverse outcome if not treated.

You will be given a set of characteristics, or a **“profile”**, for the call, including a name, birth date, address, type of insurance, and race/ethnicity. Associated medical conditions will also be included (as described above). Pretending to be the person whose profile you are assigned (or the boyfriend of the person you are assigned), you will tell the person who answers the calls at the clinic that you want to schedule your/her first prenatal visit (names of providers in the practice may be available if asked about specific providers), and that you are looking for somebody to be your regular doctor. You will seek a set of information from the clinic and record this information. If you are able to make an appointment, you will cancel the appointment before you hang up the phone (or call back immediately thereafter to cancel).

### Terminology

CASE: Each clinic is a case.

PROFILE: A profile is your insurance type, medical condition (if any), name, address, birth date – in short, your fake identity for a call. Each clinic will be called for each of the 2 patient profiles by one of the standardized patients chosen at random. That is, for a given clinical site, each of the 2 calls placed may be done by different callers.

ATTEMPT: An attempt is one call to a clinic within a particular profile. It may take several attempts to complete a profile. For example, you may call the clinic and the line is busy. Or you may call and get a voicemail saying the clinic is not open today. If this happens, you will have to call back another time, so you will have multiple attempts on the same profile.

Please try to use the words “attempt” and “profile” rather than call because it can become very confusing to distinguish between different attempts within the same profile (you are posing as the same patient still trying to get an answer from a particular clinic) and different profiles for the same clinic (you are posing as a different patient calling the same clinic you previously called under another profile).

**If you do not get through, the next attempt should be made on a different day of the week and/or in a different time slot than call attempts already made.** (It is okay to call back in the afternoon if the line is busy or the answering machine picks up in the morning.) If you talk to somebody who tells you to call a different number for the appointment desk or business office or who asks you to call back in an hour or something like that, then call when asked just as a real patient would. We are trying to behave like real patients – this is why we call back right away to a different number if asked, or try again in the afternoon if the line is busy in the morning. However, we don’t want to overestimate the number of attempts it takes to complete a profile by calling multiple times on a day when the office is closed – this is using common sense.

If you inquire about making an appointment and you are told that the services you need (e.g., opioid treatment services in pregnancy) are not available, kindly ask whether they would be able to provide a referral or contact information to a partner or affiliate provider/practice who might be able to help you. If you secure contact information for another site, it should be recorded and will be used as an additional site for sampling as well.

### Using Google Voice to Call the Clinics:

We are using Google Voice accounts to dial the clinics with believable, non-University of Utah phone numbers. To dial out to a clinic:

1. Go to google.com/voice
2. Log in with the email address/password associated with the office line you are calling from ([mountainwanderer95@gmail.com](mailto:mountainwanderer95@gmail.com) or [trailwanderer95@gmail.com](mailto:trailwanderer95@gmail.com), both with password P@RCKA2020).
3. Click the red “CALL” button in the upper left-hand corner.
4. Copy/paste or type the clinic phone number you are calling from the data spreadsheet to the “Number to call” field in Google Voice.
5. Begin recording with your audio-recording device and back-up recorder now. Be sure both devices are recording!
6. Click “Connect”.
7. Your office phone should start ringing – pick it up and the phone will automatically start dialing the clinic’s number you entered.
8. Remember: if asked for your callback number, you will reply that **you are calling from a friend’s phone and it would be best for you to call the clinic back instead.**

## Profiles of Callers

Your job is to collect information from clinics under the assumption that the patient is a particular type of person, with a particular medical condition and with a particular type of insurance. One part of your job is to maintain a consistent and appropriate persona/profile. The other part of your job is to collect the necessary information. What are the possible profiles?:

- There are **2** demographic profiles (one white/Caucasian, on American Indian/Native American) that will be used for calls placed to each of the clinic sites. Both demographic profiles share the same medical profile.
- There is **1** possible insurance profile—fee-for-service Medicaid (just getting enrolled).

### Medical Condition Profile(s)*:

You are a 22 year-old female with opioid use disorder (OUD) and have been taking buprenorphine for medication assisted treatment (M-OUD) over the last 2 years. You first became dependent on prescription opioids for chronic neck pain following a car accident several years ago, leading to heroin use. Because of life circumstances (finances, transitions, etc.) you lost access to your previous prescriber (Dr. Patel) and you ran out of your last prescription several weeks ago. You are 2 months pregnant with your second child and you would like to get an appointment as quickly as possible to establish prenatal care. You were just approved for Medicaid and your paperwork is getting processed.

You were using IV heroin during your last pregnancy, and your first child was treated in the hospital for neonatal abstinence syndrome (NAS). You went into rehab as part of your prenatal care at that time, and were started on buprenorphine and have since remained off heroin (about 2 years), except for one brief relapse 12 months ago. You got back on buprenorphine 8 months ago and have been doing well since that time. Because you lost access to your previous doctor and don’t have a current prescription, you have been pill-sharing with a friend until you can get treatment again. There is a risk you may go back to heroin without access to buprenorphine treatment.

### Insurance Profile(s):

Medicaid: You recently moved from out of state but were approved for Medicaid. You are enrolled as a Fee-for-Service member and your paperwork is being processed.

*Details of job/life situation described above are the aspects which are standardized. Additional details should normally not be needed, but if necessary, should make sense for a 22 year-old female approximately 2 months pregnant.

**Demographic Profiles**

| **Number** | **Name** | **Age** | **D.O.B.** | **Race/Ethnicity** |
| --- | --- | --- | --- | --- |
| 1 | Heather Johnson | 22 | 11/13/1997 | White/Caucasian |
| 2 | Leah Lapinski | 22 | 07/01/1998 | White/Caucasian |
| 3 | Nizhoni Yazzie | 22 | 09/25/1997 | American Indian |
| 4 | Sasha Tso | 22 | 12/27/1997 | American Indian |

##

## Scripts/Flow Chart

*****OPIOID USE DISORDER w/ MEDICATION-ASSISTED TREATMENT*****

### *Must be stated during call:*

Hi, I want to schedule a prenatal visit (or schedule a prenatal visit for my girlfriend) with [‘provider name’ if available, OR any provider].

- I am/she is new to the area. (Do NOT volunteer information about previous location/provider unless asked except as follows):
  - [If calling as Native American] I'm pregnant and need a new doctor as soon as possible. I got all my care with the Indian Health Services before. I don’t have my tribal information right now, but I could bring that to my appointment. I know my last doctor with IHS needed that.
- I am/she is pregnant. (Provide a date/approximation for last menstrual period only if asked)
- I take Suboxone and my last primary care doctor prescribed for me. Have you heard of that before?
- It is very important that my Suboxone continue while I am/she is pregnant, I/she was told that by my/her last doctor. Is that something you can help me with?
- I am out and not taking my Suboxone the way I usually do, so it’s important I see the doctor soon. [trying to see if the clerk/nurse to probe as to WHY/HOW you are not taking the medicine as you should, i.e. trying to find out what you are doing (pill-sharing) and why]
- When can I/she be seen? (Soonest available)
  - Make sure to request the specific *day* you can be seen if an appointment is offered. The *time* for appointment is not required (although it is okay if the conversation naturally flows that way)
  - If NO appointment offered:
    - What would be the normal treatments offered if they *were* accepting patients?
    - Could I speak with a nurse or doctor about my condition? I am really worried about this and need to get treatment as soon as possible.
- [specifically ask if the provider you/she will see will provide addiction treatment for her]
  - If NO or UNSURE:
    - Could I speak to a clinic nurse or medical assistant to ask about this?
    - If NO: Would you be able to refer me to someone that would be able to help me with this? (If offered a referral, obtain any available contact info and contact the referral site using the information you were given)

### *If asked:*

***What is the appointment for?***

I need/she needs prenatal care and need someone to prescribe my/her Suboxone while I am/she is pregnant. I know that I/she won’t be able to handle it if I/she can’t get the treatment I need/she needs.

***What are you taking? How much are you taking? How much do you have left?***

- I take/she takes Suboxone
- 8mg twice per day
- I am out so I have been taking it differently
  - *If probed*: I have been sharing pills with my friend
    - *If probed*: Because I was transitioning, missed my appointment, and my doctor cut me off. I had to get new insurance here in Utah so now trying to get my prescription again.

***Have you ever used illicit opioids or other drugs?***

I/she used to use heroin but stopped during my/her last pregnancy. One brief relapse a year ago but doing well since that time. My/Her first child was treated for “addiction” (neonatal abstinence syndrome) and child protective services was involved. I’m worried about that with this pregnancy.

***Do you have any other symptoms or medical conditions?***

Not really, I don’t think so.

***Do you take any medications/other medications?***

I/she has been taking Suboxone (buprenorphine), but different than I normally do.

- - *If probed:* I am sharing pills with a friend.
  - *If probed on loss of prescription:* I/she lost my/her coverage but am/is getting Medicaid now; **OR** My last prescription from the last provider expired and I have no money to pay for the prescription.

I/she started taking a prenatal vitamin when I/she found out I/she was pregnant.

I/she am/is taking Zoloft.

- *If asked about depression symptoms/severity*: Yeah, it has been pretty bad lately. I think I am getting in a bad spot.
- *If asked about prior suicide attempts*: Yeah, I took a bunch of pills years ago. I don’t think I would do that again, but I do feel like I am getting into a bad spot again.
- *If asked about suicidality now:* no, I don’t feel that way right now. (no thoughts of suicide at all)

***Do you have a naloxone/overdose emergency kit?***

- No, I’ve never received one of those.

***Do you have health insurance?***

I/She was approved for Medicaid in Utah, and the paperwork is being processed.

***Where are you moving from/where did you get your care previously?***

I/She just moved from the Denver (if white/Caucasian)/Window Rock (if American Indian) area. I/She saw Dr. Patel (you can’t remember her first name; too hard to pronounce or spell), who prescribed my/her Suboxone and helped me/her with my/her pregnancy.

**CANCEL THE APPOINTMENT IF YOU HAVE ONE!!!**

***If you are unable to cancel an appointment before getting off the phone, call right back and cancel.**

### *Flow Chart:*


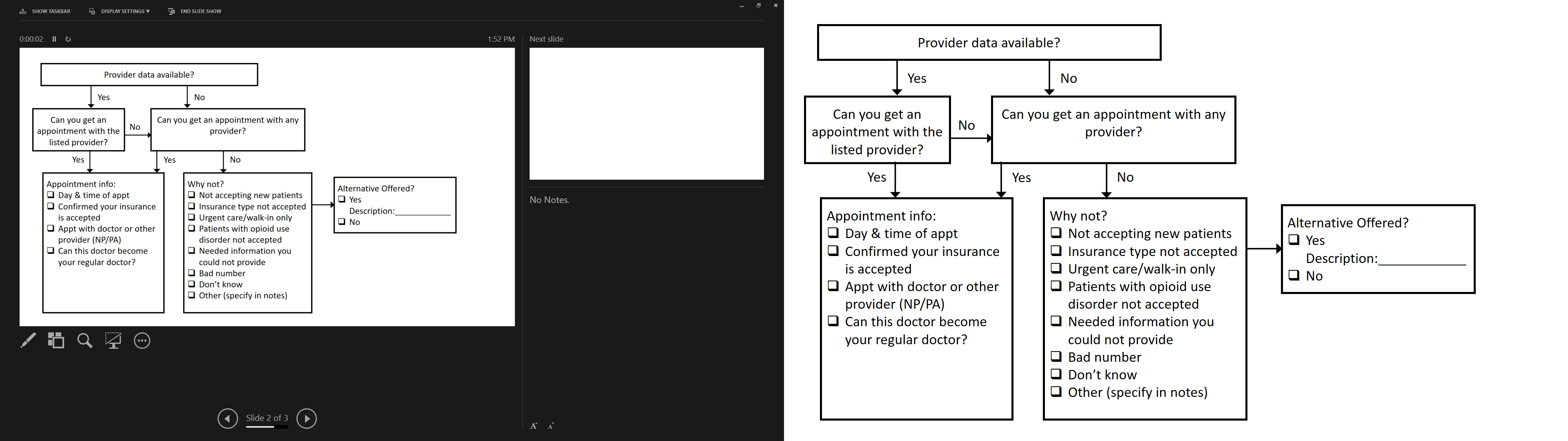


## Work-Arounds

*The most important piece of information we are trying to collect is whether or not the clinic will provide the treatment you are seeking and what resources are offered if the clinic cannot or will not provide treatment.* A problem with this approach is that clinics typically want new patients to register, and may require registration before giving us an actual appointment date. To register us as patients, they may ask for information like address, phone, birth date, social security number, and insurance information. Some of this we can provide (you will be assigned an address, name and birth date, for example) and some of this we cannot provide (it is a federal offense to give out a false social security number so we NEVER do this).

Here we provide you with some “work-around” scripts to help you try to avoid or evade these questions. However, we cannot anticipate every possible situation and you will need to do your best to handle the questions you are asked within the profile you are assigned. Try not to give up before you know whether the patient can be scheduled, when the appointment will be if so, and any contact information for referrals is obtained if not. Other information is secondary. We ideally want to avoid the “creation” of accounts in schedulers’ systems as much as possible to minimize the burden of administrative time we consume from each in the research process.

Work-Arounds (written from the perspective of the patient, but can be adapted to the perspective of the boyfriend):

### Not accepting new patients due to COVID-19

- Would you be able to tell me what you would normally do, once COVID-19 improves? I am just trying to figure out where I should plan to get my care, and this clinic would be the most convenient.
- Where have you been referring new patients to right now? I think my condition is urgent.
- Yeah, I know it’s been pretty crazy. Could I speak with one of your nurses, medical assistants, or doctors about my situation? This is actually kind of urgent, so I would like to talk with a medical person to figure out what I can do.

### Not doing face-to-face visits due to COVID-19

- If I can’t be seen but can have an appointment with the doctor on the phone, that is probably ok. When could I set up a phone call?
- *If video visit offered*: I’ve never done that before, but if you are able to help me set it up, I might be able to do it. I don’t have access to the internet, currently.

### Medicaid

What is your account number? [Or any other info from your insurance card they want]

- I don’t have the card on me right now.

If asked to call back with card information:

- Would you be able to give me a few of the first available dates to check here at work before I go home at lunch (or whenever) to get my card? That way I can schedule the appointment when I call you back later today.
- I don’t have the card yet. I just called my social worker to start my insurance again. I didn’t ask what the number was. Can I call you back after I make the appointment?

If told that you are not in the system [i.e., receptionist puts your name and birth date in to look for Medicaid account number]:

- I just joined within the last few days. Could that be why I am not yet in the system? Can I go ahead and schedule the appointment and just bring my card with me when I come?
- My caseworker said it should be active by the 1^st^ of the month. Can you tell me when first available appointments are right now and I will call back once it’s activated?

If asked whether you are registered or assigned to Dr. [last name from spreadsheet]:

- I am not sure if I’m registered yet but that’s who my friend said I should see. Can I schedule an appointment? [Typically, a case worker would instruct you how to register with that doctor.]
- I haven’t been assigned yet, and I wanted to check who would be first available before I made that decision.

*If told that you will need to register or make sure the physician is assigned as your PCP prior to your appointment, assure the receptionist that you will.*

### Insurance

What is your health insurance?

- I know it’s Medicaid. I think I can go anywhere they take Medicaid.
- I don’t have my card with me right now, but they said your clinic was on the list. I can bring it when I come in.

We don’t take that insurance plan.

- Which plans do you accept? [**Record alternate plan in notes and call back another day.**]

### Other Work-Arounds

Preferred Location

- Always make the appointment at the location **listed in the spreadsheet**, even if there is an earlier appointment time for another location in the network of practices. Referrals to other clinics/sites will be coded as a separate attempt (to a referral site).

We no longer accept that type of Medicaid.

- Can you tell me what plans you do accept? (The insurance is new; I may have gotten the name wrong – when I looked this clinic up online, I thought I saw my plan listed).
- You can also Google the clinic/doctor and try to find a list of accepted plans.

Is the plan through your employer or through “Obamacare”/the health insurance exchange/the Marketplace?

- Say you are not employed and are in the process of getting Medicaid

They won’t give you a specific date (e.g., must be accepted first as a patient)

- I understand I may not get that appointment, but I am looking at a couple different clinics and want to see which one I can get in with soonest.

Central Scheduling System: “You are already in our system – did you used to live on _____?”

- No, that must be someone else. I never lived at that address.

Social Security Number

- I don’t know it off-hand, but I can bring it in when I come in.
- I don’t like giving it out over the phone. I’ll bring it when I come in.
- I don’t have my social security number memorized, but I will find my card and bring it when I come in.

What part of the city do you live in?

- Well, I just moved here so I’m not altogether clear about directions yet. My address is [faux address from spreadsheet] *OR* I’m not sure what the address is where I will be staying, but I can call back when I get it. *OR* I’m staying with a friend right now and haven’t moved into my place yet. I can get that information for you and call you back.

What is your race/ethnicity? [if asked during registration process]

- White [or Native American]

Who is your employer?

- I am not working right now.

Why did you pick this doctor?

- He/She was on my insurance plan’s list of covered providers.
- My friend recommended him/her.

Somebody will call you back later.

- Actually I’m on someone else’s phone – can you give me a time when I can call you back?

[**Avoid having the scheduler call you back. Always ask for a day/time for YOU to call back.**]

You need to have lab tests before we can prescribe you Suboxone.

- No problem, when can I come in to have them done?

You need to be accepted as a new patient before we can schedule an appointment.

- Instructions: Complete the new patient questionnaire if it is over the phone and provide the information for your “profile”. Ask for a time to call back to see if you have been accepted as a new patient and schedule your appointment. *Be sure to keep careful note of which information you provide to this office for when you make your follow-up call!*
- If told that you must come in to complete the new patient questionnaire, ask if you are able to come in this afternoon during your lunch (check if the office is closed for lunch at any time). AND could you give me a few dates of first available appointments to check before I leave work – that way I could schedule after I finish the paperwork?

Who was your last doctor?

- I was seeing Dr. Patel before [you don’t remember his first name, and only know he was in Denver]. I have my old records at home, if it is necessary I think I can find out from them.

You need to release your previous patient records to us before we can schedule an appointment.

- Say that you haven’t seen a doctor a few months, but you do have your previous medical records. Could you bring them in yourself?
- I lost access to my doctor in [previous location] and I no longer have my phone so I don’t have his number saved. I know his name was Dr. Patel. Can we move forward in scheduling the appointment? 🡪 If pressed, say you will look for the contact information. Call back on another day, state that you looked through your records and online and could not locate your last doctor’s contact information, and ask if you can move forward with scheduling an appointment.

If told that records release or any other task is required before scheduling the appointment:

- I will do my best to go through the process (find my card, straighten out my insurance with my caseworker/employer, come in to fill out the paperwork, etc.) as soon as possible. If I am able to get that taken care of today, when is the next available appointment I could get? I understand you can't guarantee that appointment date until you have all the required information and/or the physician accepts me, but I would like to have an idea of when it might be because I need to plan for [my kid’s schedule, arrange transportation, family members’/friends’ work schedules]."

You will need to fill out a questionnaire before the appointment and we will email it to you.

- Sure, my email address is [mountainwanderer95@gmail.com](mailto:mountainwanderer95@gmail.com) (password is “P@RCKA2020”) OR [trailwanderer95@gmail.com](mailto:trailwanderer95@gmail.com) (password is “P@RCKA2020”).

## Data Collection

### Post-visit questionnaire

At the conclusion of each call, you will need to complete a short post-visit questionnaire using REDCap (<https://j.mp/2V1CIx3>). The content of this questionnaire is outlined in the Fields for Data Collection section below.

### Coding of recorded audio

In addition to post-visit questionnaires, each call will also be audio-recorded, and these recordings will be separately reviewed and coded for specific information, as outlined in the Fields for Data Collection section below.

### Data entry best practices

1. *Entering dates*: the date fields will be in plain text format, so enter dates without dashes or slashes:

ex. For April 1, 2014, enter “040114”

1. *Unclear or unanswered responses*: if the answer to a question is unclear or unanswered, please indicate it as such. We want to be as precise as possible.
2. *Numeric fields*: in fields with numbers/numeric codes, do not include any text or symbols (including <, >, ?, etc.) as the statistical software can’t analyze anything but exact numbers.
3. *Varying experience by call*: if you are inadvertently cut-off mid-call when they tried to transfer you to another line and then got a different person who provided more help and information than the first person had, record the most helpful response because a real patient would have called right back and could have gotten the same improved help from the second person.
4. *Record everything*: first, write any notes that will help you (or others) on subsequent calls – this may include unforeseen barriers in calling, quirks of the appointment system, unusual clinic hours/scheduling ability, or new work-arounds that need to be developed. Also, record any information that might at all be relevant to the study such as side comments made by the scheduler to you or others – we will only have the audio recording, so subjective impressions must be written down to be included in the analysis! Always err on the side of assuming it will be important.

# Table S2. Disclosure protocol

| *Voluntarily disclose*   - Pregnancy status - Diagnosis of opioid use disorder - Use of Suboxone (don’t say why you take it, just that you take it) - “I’m taking my Suboxone differently than I normally do” - Need for appointment as soon as possible - [For American Indian/Native American profile only] recent relocation from reservation and prior care with Indian Health Service | *Voluntarily inquire*   - Willingness of clinic to accept you as patient for prenatal care - Familiarity with Suboxone (has heard of it or not; knows what it’s for) - Willingness of clinic to provide care for SUD/prescribe controlled substance (Suboxone) - Date for next available appointment - Degree of experience clinic has in managing patients with conditions similar to yours - Referral to another provider if clinic unwilling/unable to offer appointment and/or treat OUD |
| --- | --- |
| *Disclose in response to general inquiry or if lack of disclosure impedes obtaining desired information*   - Duration of pregnancy/last period - Prior heroin use/alcohol use & withdrawals - Hospitalization of first child for neonatal abstinence syndrome - Prior involvement of child protective services and your concern about that for new pregnancy - Information about previous provider who managed patient’s SUD - Medical history   - Medications (Suboxone, Zoloft, prenatal vitamin), chronic neck pain | *Disclose only in response to specific inquiry*   - Pill-sharing with friend due to lack of access to prescriber - Diagnosis of depression, prior suicide attempt - When last prescription ran out/will run out - Concerns about transportation to appointment - Lack of naloxone/overdose kit - Alcohol use history (no history of significant alcohol use) |
| *Do not disclose*   - Telephone call-back number (using a friend’s phone, no personal phone at this time) - Address (may disclose address for face-to-face visit) - Other detailed personal info (no SSNs, no prior employment info) | |

# Table S3. Sampled clinics by type

|  | County 1 | County 2 | County 3 |
| --- | --- | --- | --- |
| Clinics | 8 | 3 | 6 |
| Primary Care | 6 | 3 | 6 |
| OB/GYN | 2 | 0 | 0 |

# Figure S1. Telephone Appointment Request and Face-to-face Visit Algorithms for Unannounced Standardized Patients with Pregnancy and Opioid Use Disorder.

1. **Telephone Appointment Request**


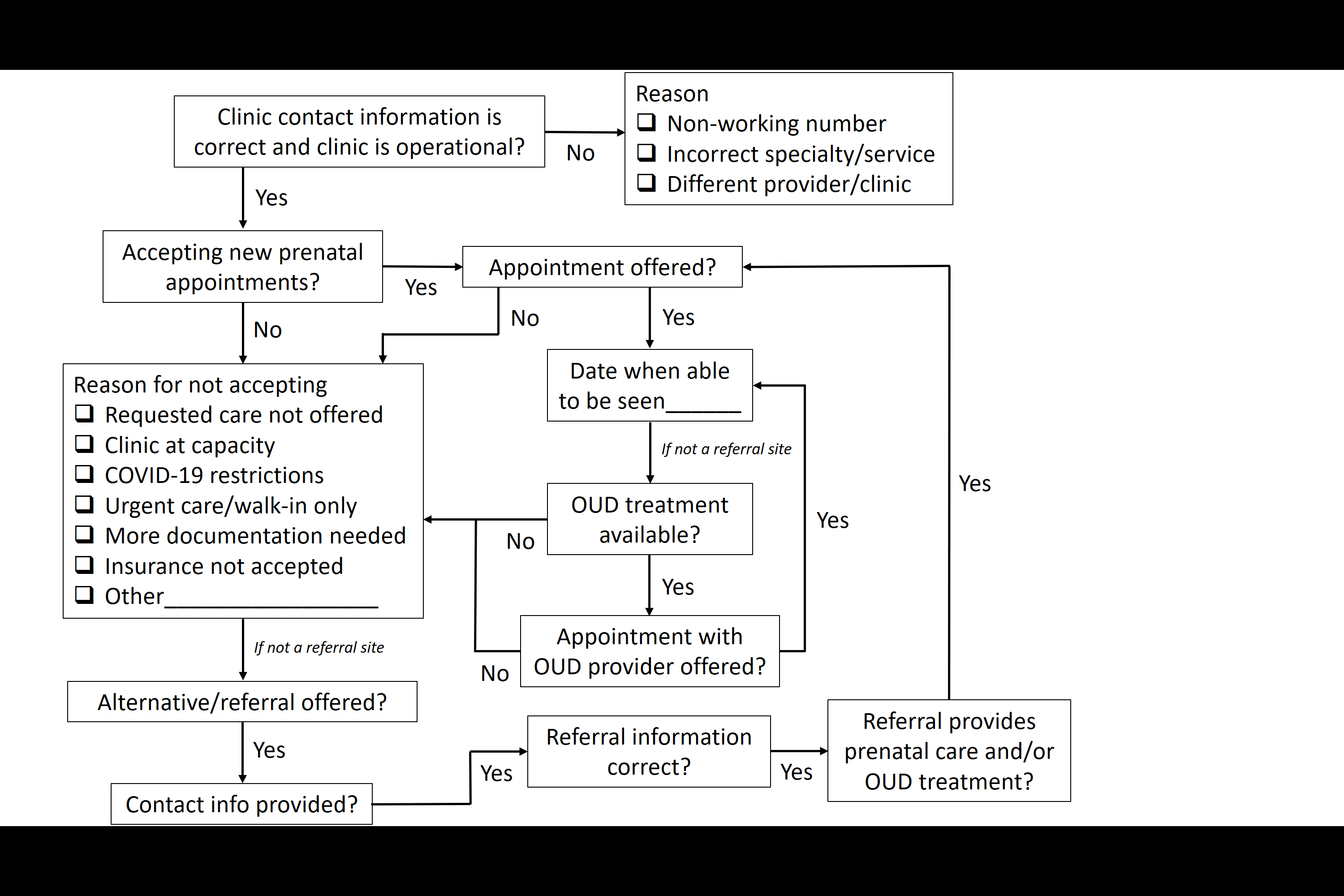


**b) Face-to-face Visit**


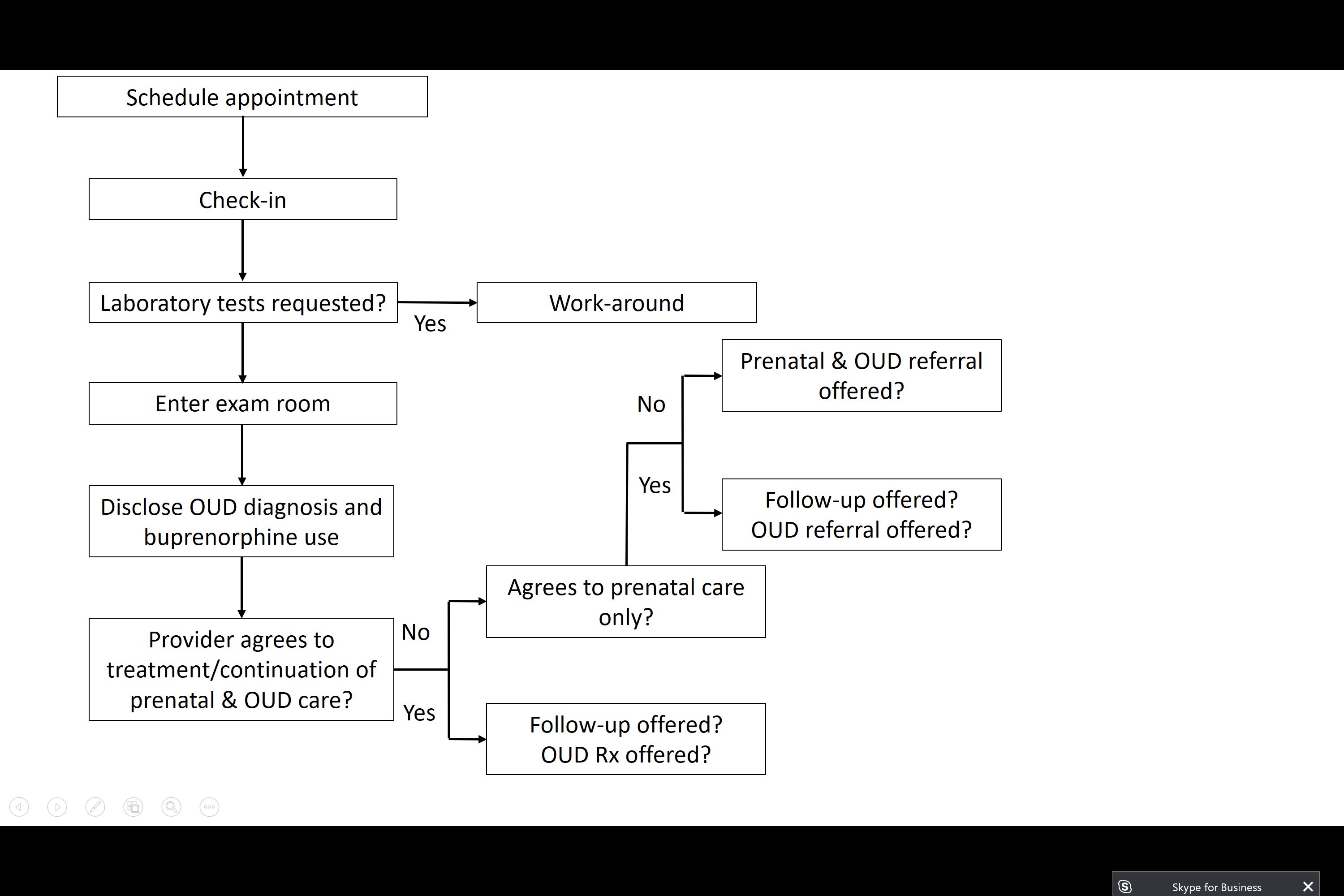

Supplement: Supplementary file 1 — Additional file 1. (1) Summary of evidence-based guidelines for opioid use disorder in pregnancy; (2) unannounced standardized patient training guide; (3) unannounced standardized patient disclosure protocol; (4) summary of study sample; and (5) unannounced standardized patient encounter algorithms. [file 13722_2021_246_MOESM1_ESM.docx]
